# *p*-Anisaldehyde Exerts Its Antifungal Activity Against *Penicillium digitatum* and *Penicillium italicum* by Disrupting the Cell Wall Integrity and Membrane Permeability

**DOI:** 10.4014/jmb.1911.11032

**Published:** 2020-03-09

**Authors:** Jinxin Che, Xiumei Chen, Qiuli Ouyang, Nengguo Tao

**Affiliations:** 1 School of Chemical Engineering, Xiangtan University, Xiangtan 405, Hunan, P.R. China; 2 Postdoctoral Station of Chemical Engineering and Technology, Xiangtan University, Xiangtan 411105, Hunan, P.R. China

**Keywords:** *p*-Anisaldehyde, antifungal activity, *Penicillium digitatum*, *Penicillium italicum*, inhibitory mechanism

## Abstract

*Penicillium digitatum* and *P. italicum* are the two important postharvest pathogens in citrus, causing about 90% of the total loss of citrus fruit during storage and transportation. Natural fungicides such as essential oils have been widely used instead of chemical fungicides for preventing and controlling postharvest diseases. In this research, *p*-anisaldehyde exhibited a strong inhibitory effect on *P. digitatum* and *P. italicum*, with the minimum inhibitory concentration and minimum fungicidal concentration values of both being 2.00 μl/ml. Additionally, *p*-anisaldehyde visibly inhibited both the green mold and blue mold development of citrus fruits inoculated with *P. digitatum* and *P. italicum*. The mycelia morphologies of these pathogens were greatly altered, and the membrane permeability and cell wall integrity of mycelia were severely disrupted under *p*-anisaldehyde treatment. These results suggest that the antifungal activity of *p*-anisaldehyde against *P. digitatum* and *P. italicum* can be attributed to the disruption of the cell wall integrity.

## Introduction

Citrus fruit is one of the most commercially important agricultural products in the world [1], with global production exceeding 140 million tons in 2016 (FAO, 2016). The susceptibility of citrus to postharvest diseases results in wastage and deterioration and causes serious economic losses, and most of these diseases are caused by fungal pathogens [2, 3]. *Penicillium digitatum* and *P. italicum* are the main pathogens of two important postharvest diseases known as green mold and blue mold, which cause about 90% of the total economic loss of citrus fruit during storage and transportation [4, 5]. Nowadays, many chemical fungicides are being widely used to control these postharvest diseases and these include fludioxonil, imazalil, and pyrimethanil [6]. Nevertheless, with the long-term and repeated use of such fungicides, their effectiveness has been gradually weakened by the proliferation of resistant fungus strains. Moreover, consumers are becoming increasingly concerned over food safety and residual contamination by fungicides [7, 8]. In this regard, plant essential oils as natural fungicides are generally recognized as safe. Over the past decade, many such essential oils could be considered as biodegradable and safe, natural alternatives to chemical fungicides due to their broad spectrum of fungicidal activities against postharvest pathogens [9-11].

Among the essential oils, *p*-anisaldehyde is a naturally occurring, fragrant phenolic compound that is primarily isolated from anise, cumin, fennel, and garlic [12, 13], which has been widely used for the production of antimicrobial drugs in the pharmaceutical industry [14]. In recent studies, *p*-anisaldehyde showed strong antimicrobial activity against many microorganisms including *Staphylococcus aureus* [15], *Candida* [12], and *Saccharomyces cerevisiae* [16]. However, knowledge about the antifungal activity of *p*-anisaldehyde against *P. digitatum* and *P. italicum* in citrus fruits was rather limited.

The purpose of this study was to investigate the antifungal efficiency of *p*-anisaldehyde on the mycelial growth of *P. digitatum* and *P. italicum* and to reveal the possible mechanism involved.

## Materials and Methods

### Microorganisms, Fruits and Chemicals 

*P. digitatum* (Pds-01) and *P. italicum* (Pis-01) strains were isolated from infected *Satsuma mandarin* fruit (*Citrus unshiu Marc*) and preserved in the Department of Biotechnology and Food Engineering, Xiangtan University, Hunan, China.

Fruits of *Satsuma mandarin* (*Citrus unshiu Marc*. cv. Miyagawa Wase) used for in vivo assay were picked in a local orchard near Xiangtan University, China, in October 2018. The citrus fruits for experiment were defect-free and with similar size and the same maturity level.

*p*-Anisaldehyde (>98%) was purchased from Aladdin (China). Propidium iodide and an alkaline phosphatase (AKP) kit were purchased from Solarbio Science and Technology Co., Ltd., Beijing, China. Commercial wax (SP-1) was obtained from Bo Cheng Chemical Engineering Co., Ltd (China). Other chemicals were of analytical grade and purchased from Xilong Chemical Co., Ltd. (China).

### Effects of *p*-Anisaldehyde on Mycelial Growth

The effects of *p*-anisaldehyde on the mycelial growth of *P. digitatum* and *P. italicum* were evaluated by the agar dilution culture method [4]. The culture medium contained 20 ml potato dextrose agar (PDA) supplied with final concentrations of *p*-anisaldehyde (0.00, 0.12, 0.25, 0.50, 1.00, 2.00 and 4.00 μl/ml). A mycelial agar plug (diameter = 6 mm) from a 7-day-old culture of *P. digitatum* or *P. italicum* was inoculated into the PDA culture above. The plates were incubated for 5 d at ambient temperature (24 ± 1°C). The colony zone diameters were measured per 24 h. All tests were performed in triplicate.

The inhibitory rate of *p*-anisaldehyde was calculated as follows:

(1) $$\mathrm{Inhibitory}\mathrm{rate}(\%)=\frac{\mathrm{Diameter}\mathrm{of}\mathrm{CK}–\mathrm{Diameter}\mathrm{of}\mathrm{treatment}}{\mathrm{Diameter}\mathrm{of}\mathrm{CK}}\times 100\%$$

The lowest concentration that completely prevented the growth of *P. digitatum* or *P. italicum* after 2 d of cultivation was considered as the minimum inhibitory concentration (MIC). The minimum fungicidal concentration (MFC) was defined as the concentration that completely inhibited pathogen growth after 4 d [17].

### Effects of *p*-Anisaldehyde on Disease Incidence

Citrus fruits were treated according to the previously described method [18]. In detail, citrus fruits were divided into 3 groups. Each group was composed of three repeats and each repeat included ten citrus fruits. For each surface-sterilized citrus fruit, two wounds (length of 5 mm and depth of 5 mm) were made around the fruit equator evenly using a scalpel, with each wound subsequently inoculated with 20 μl spore suspension, and left to air-dry. The *P. digitatum* and *P. italicum* spore suspensions were adjusted to 1 × 10^8^ CFU/ml. The inoculated fruits were sprayed with *p*-anisaldehyde wax at MFC or 10×MFC, then left to air-dry. The CK was sprayed with wax only. The treated fruits were stored in plastic boxes at 25 ± 2°C with 85~90% relative humidity, and the disease incidence was measured each day. The inoculated fruits with wax were used as a control.

The disease incidence rate was measured by counting the number of rotting wounds using the following formula:

(2) $$\mathrm{Decay}\mathrm{incidence}\mathrm{rate}(\%)=\frac{\mathrm{Number}\mathrm{of}\mathrm{rotten}\mathrm{wounds}}{\mathrm{Number}\mathrm{of}\mathrm{total}\mathrm{wounds}}\times 100\%$$

### Scanning Electron Microscopy (SEM) 

The SEM analysis was performed according to our previous method [17]. The mycelia of *P. digitatum* and *P. italicum* were obtained from 2-day cultures grown in potato dextrose broth (PDB) at 25 ± 2°C, 150 rpm. These were treated with *p*-anisaldehyde at 0, 1/2 MIC and MIC for 2 h, respectively. The mycelia morphologies of the samples were directly obtained with a JSM-6610LV scanning electron microscope (JEOL, Japan).

### Membrane Permeability Assays

The membrane permeability of mycelia was analyzed by the propidium iodide (PI) staining method [19]. Two-day-old mycelia from PDB were collected and treated with *p*-anisaldehyde (0, 1/2 MIC, and MIC) for 30, 60, and 120 min. The treated mycelia were dyed with 10 μg/ml PI for 5 min at 30°C, then centrifuged at 4,000 ×g for 15 min after being washed three times with PBS. The mycelia were photographed with an Eclipse TS100 epifluorescence microscope (Nikon Corporation, Japan). The fluorescence values were determinated by a F97 PRO fluorescence spectrophotometer (Lengguang Technology, China) with an excitation wavelength of 535 nm and the emission wavelength at 615 nm. The results were expressed as the fluorescence value folds.

### Determination of Extracellular Alkaline Phosphatase (AKP) Activity

Two-day-old mycelia from PDB were collected and treated with *p*-anisaldehyde (0, 1/2 MIC, and MIC) for 30, 60, and 120 min. The extracellular AKP activity was determined by a UV-2450 UV/Vis spectrophotometer (Shimadzu Co., Ltd., China) according to the instructions for the Alkaline Phosphatase (AKP/ALP) Assay Kit (Beijing Solarbio Science & Technology Co., Ltd., China). All of the tests were performed in triplicate.

### Statistical Analysis

Each parameter was performed in triplicate. All data were analyzed using the SPSS statistical software (Version 16.0) (SPSS Inc., USA). The data were expressed as the mean ± SD (standard deviation) by measuring three independent replicates and analyzing them by the least significant difference (LSD) test of variance (ANOVA). A value of *p* < 0.05 was considered statistically significant.

## Results

### Effects of *p*-Anisaldehyde on Mycelial Growth

The effects of *p*-anisaldehyde on the mycelial growth of *P. digitatum* and *P. italicum* are shown in Tables 1 and 2. The in vitro assay results on agar plates indicate the mycelial growth of *P. digitatum* and *P. italicum* was markedly (*p* < 0.05) inhibited in a dose-dependent manner by *p*-anisaldehyde. After 5 d of culture, about 11.5 ± 2.8% and 34.5± 10.0% of the *P. digitatum* and *P. italicum* mycelia growths were inhibited by 0.12 μl/ml of *p*-anisaldehyde. When the concentration of *p*-anisaldehyde reached 2.00 μl/ml, the mycelia showed no visible growth until 4 days of culture. Therefore, the MIC and MFC of *p*-anisaldehyde against *P. digitatum* and *P. italicum* were both estimated to be 2.00 μl/ml.

### Effects of *p*-Anisaldehyde on Disease Incidence

The ability of *p*-anisaldehyde to inhibit the green mold of citrus fruit inoculated with *P. digitatum* is shown in Table 3. The fruit in control group began to rot after 3 d of storage, and the disease incidence was significantly (*p* < 0.05) delayed in *p*-anisaldehyde treatment in a dose-dependent manner. The control fruit disease incidence was 30 ± 10% at 5 days of storage, whereas this percentage of disease incidence in citrus fruits treated with *p*-anisaldehyde (MFC) was reduced to 10 ± 10%. With prolonged storage time, the disease incidence of green mold increased. After 7 days of storage, the green mold incidence of control fruits was 100% while that of *p*-anisaldehyde-treated (1 × MFC and 10 × MFC) fruits was only 90 ± 8.6% and 45 ± 5%, respectively.

Similarly, the blue mold development of citrus fruit inoculated with *P. italicum* was inhibited by *p*-anisaldehyde treatment (Table 4). The control fruit began to rot after 1 d of storage. After 8 days of storage, the disease incidence of the control fruits was 35 ± 0%, and it was still 0 ± 0% in citrus fruits treated with *p*-anisaldehyde (MFC and 10 × MFC). The blue mold incidence in the control fruits was 100% after 12 days of storage, and the incidence of *p*-anisaldehyde-treated (MFC and 10 × MFC) fruits was only 25 ± 10% and 5 ± 0% respectively. The inhibitory effect of *p*-anisaldehyde on blue mold incidence was higher than that on green mold.

### Scanning Electron Microscopy

**Fig. 1:**
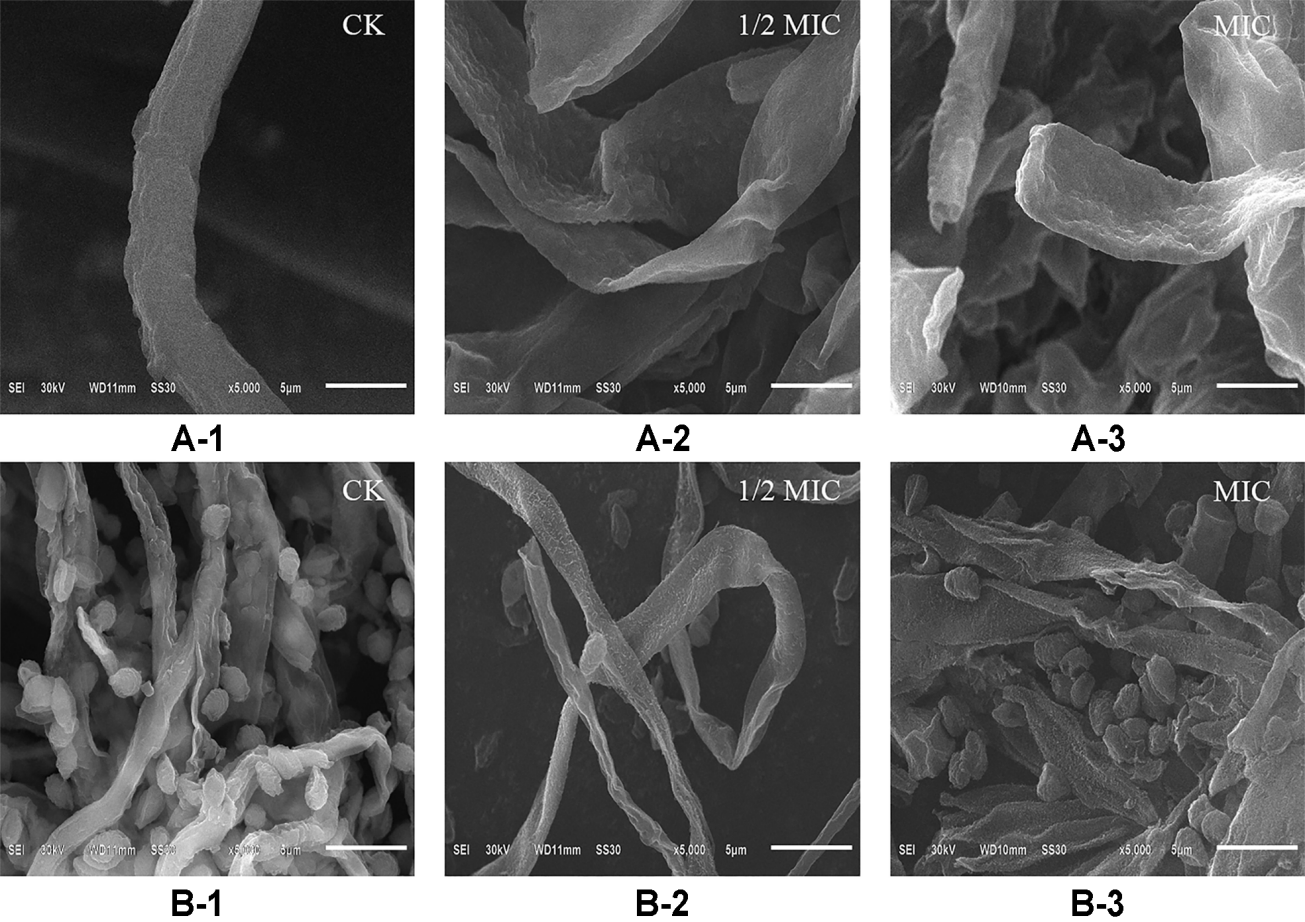
SEM images of *P. digitatum* (A-1, A-2, A-3) and *P. italicum* (B-1, B-2, B-3) mycelial morphology after *p*-anisaldehyde treatment. Untreated hyphae (**A-1, B-1**); treatment with 1/2 MIC of *p*-anisaldehyde (**A-2, B-2**); treatment with MIC of *p*-anisaldehyde (**A-3, B-3**) (bar = 5 μm).

**Fig. 2:**
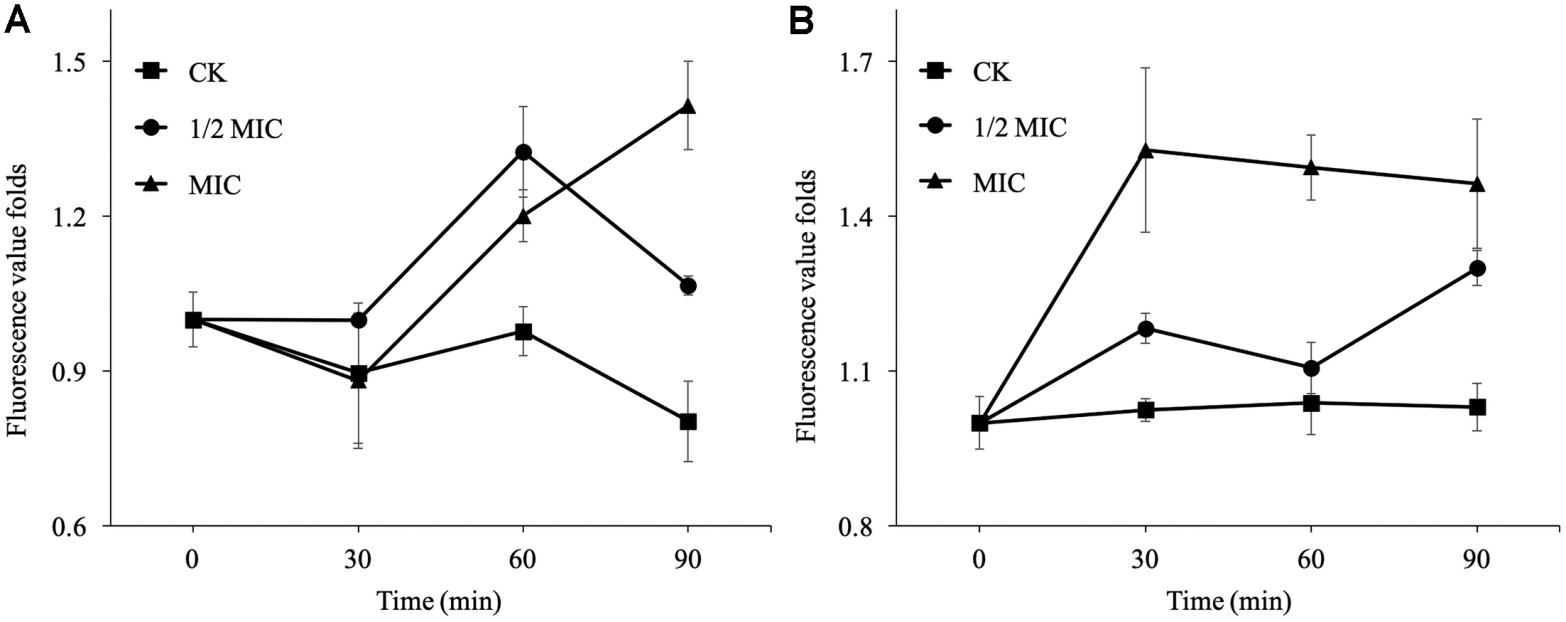
Effects of *p*-anisaldehyde treatment on the PI fluorescence fold changes of *P. digitatum* (A) and *P. italicum* (B) mycelia. Data presented are the means of the pooled data. Error bars indicate the SDs of the means (*n* = 3), the same as below.

**Fig. 3:**
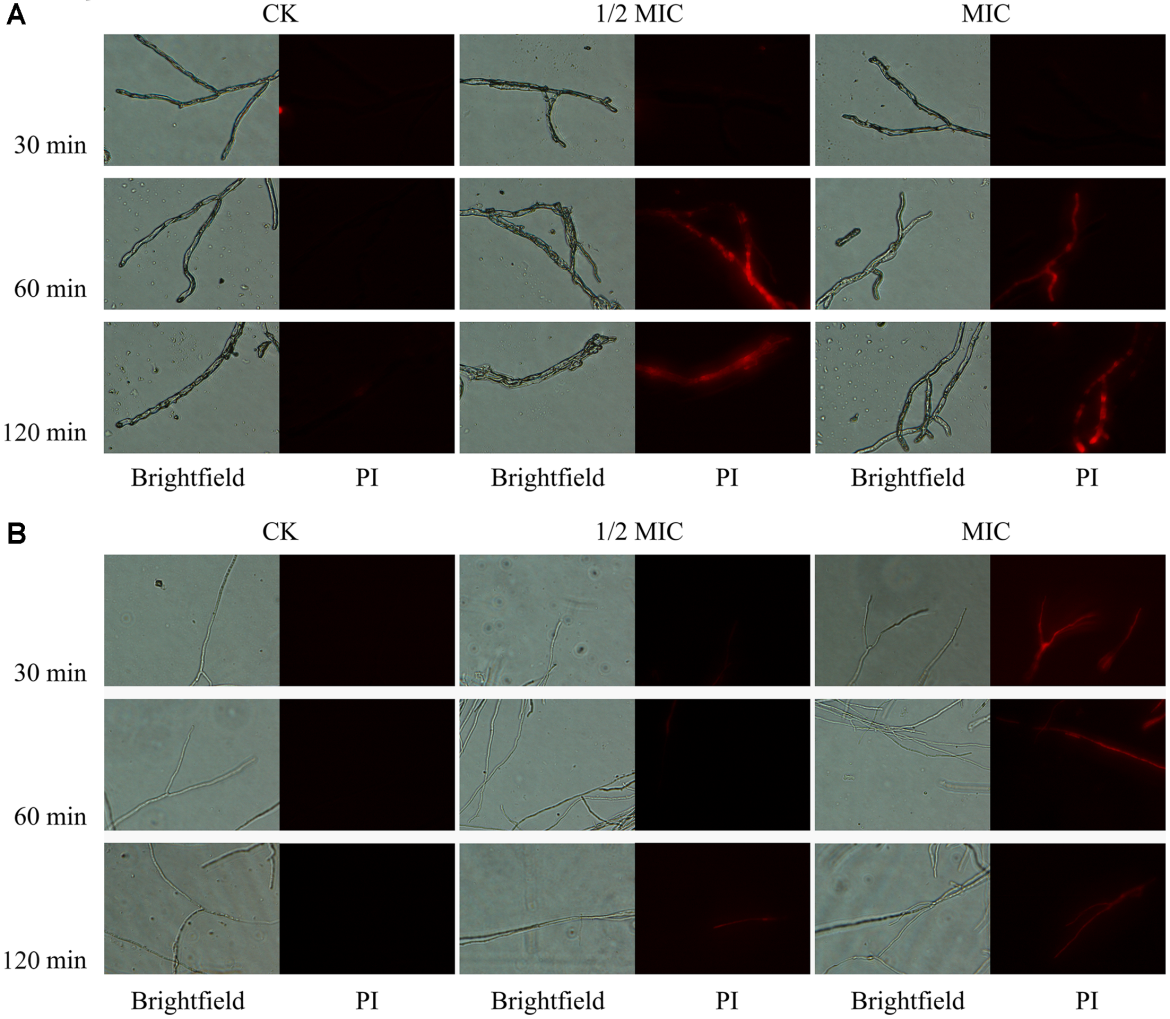
Effects of *p*-anisaldehyde treatment on the plasma membrane integrity of *P. digitatum* (A) and *P. italicum* (B) mycelia.

The effects of *p*-anisaldehyde on the morphology of *P. digitatum* and *P. italicum* mycelia were examined by SEM (Fig. 1). As shown in Figs. 1A-1 and 1B-1, the control samples had a tubular, regular, full shape, with homogeneous and smooth surface morphology. In contrast, the 1/2 MIC of *p*-anisaldehyde-treated mycelia showed irregular, wizened, rough and collapsed surfaces (Figs. 1A-2 and 1B-2). This phenomenon was more apparent as the concentration of *p*-anisaldehyde increased. As revealed by Figs. 1A-3 and 1B-3, the MIC *p*-anisaldehyde-treated mycelia became flat, widened, and wrinkled with irregular structures and shapes.

### Membrane Permeability Assay

The effects of *p*-anisaldehyde on the membrane permeability of *P. digitatum* and *P. italicum* are shown in Figs. 2 and 3. After 30 min of exposure, the fluorescence intensities of all treatments in *P. digitatum* samples were decreased (Fig. 2A). However, the fluorescence values in *P. digitatum* treated with 1/2 MIC and MIC of *p*-anisaldehyde were 1.32 ± 0.09 and 1.20 ± 0.05 times that of the control at 60 min of incubation. With time prolonged to 120 min, the fluorescence value of *P. digitatum* in MIC *p*-anisaldehyde treatment was nearly 1.41 ± 0.09 times higher than that of the control. With regard to *P. italicum*, the fluorescence values of the *p*-anisaldehyde-treated mycelia were significantly higher (*p* < 0.05) than control groups during the whole period (Fig. 2B). The fluorescence values of the MIC *p*-anisaldehyde-treated *P. italicum* were approximately 1.53 ± 0.16, 1.49 ± 0.06, 1.46± 0.12 times higher than that of control at 30, 60, and 120 min of exposure.

The fluorescence microscopy images of the mycelium treated with *p*-anisaldehyde are shown in Fig. 3. The results were consistent with fluorescence values described above. There was no visible red fluorescence observed in CK after 120 min of incubation. In the *P. digitatum* mycelium treated with 1/2 MIC and MIC, red fluorescence was exclusively observed after 60 min of exposure (Fig. 3A). And in the *P. italicum* hyphae treated with MIC, the red fluorescence was observed after 30 min of exposure (Fig. 3B).

### Determination of Extracellular Alkaline Phosphatase (AKP) Activity

**Fig. 4:**
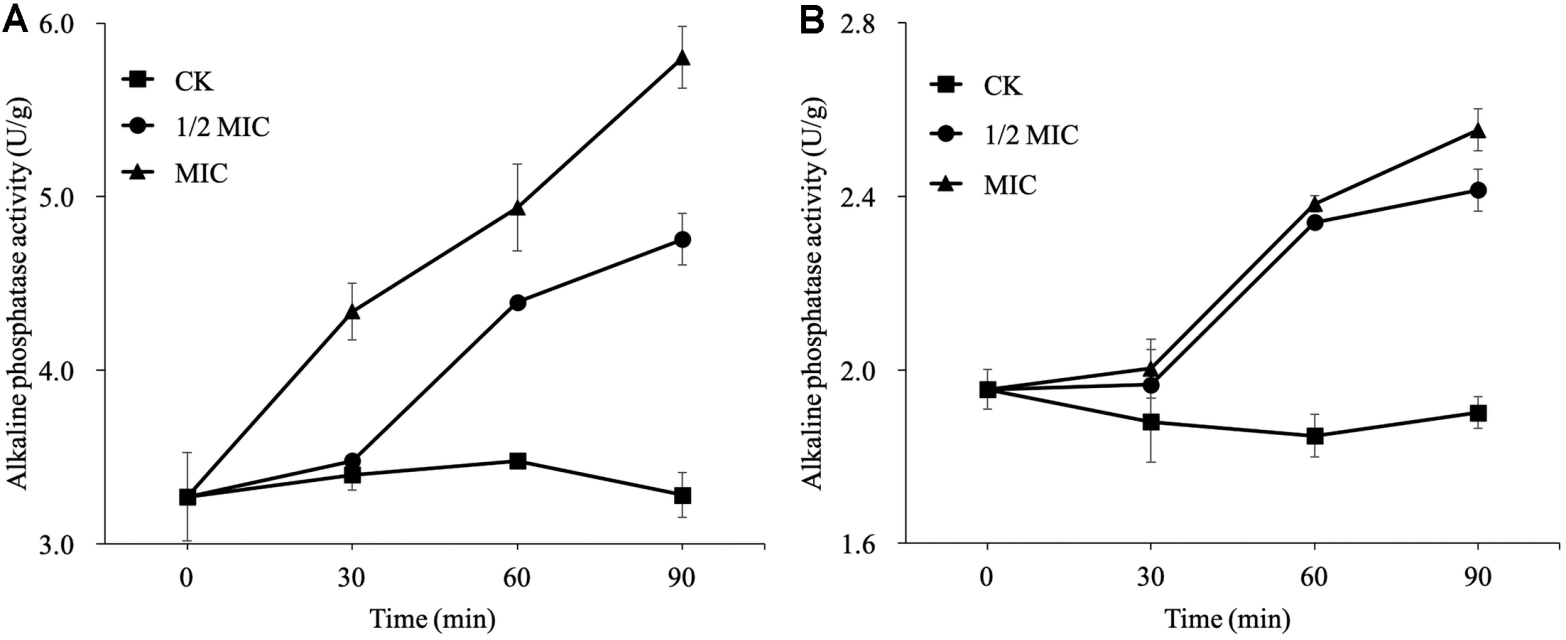
Effects of *p*-anisaldehyde treatment on the AKP activity of *P. digitatum* (A) and *P. italicum* (B).

The effects of *p*-anisaldehyde (0, 1/2 MIC and MIC) on the extracellular AKP activity of *P. digitatum* and *P. italicum* were shown in Fig. 4. The extracellular AKP activity in 1/2 MIC and MIC *p*-anisaldehyde-treated *P. digitatum* and *P. italicum* mycelia were all higher than that of control. The extracellular AKP activity in MIC *p*-anisaldehyde-treated *P. digitatum* was higher than that of 1/2 MIC treatment during the whole period. After 120 min of treatment, the extracellular AKP activity of *P. digitatum* mycelia treated with MIC of *p*-anisaldehyde was 5.80 ± 0.18 U/g, which was significantly higher than the control (3.27 ± 0.13 U/g) (Fig. 4A). The extracellular AKP activity levels of both 1/2 MIC- and MIC-treated *P. italicum* showed the same variation trend during the whole incubation period (Fig. 4B). The extracellular AKP activity of *P. italicum* treated with 1/2 MIC and MIC *p*-anisaldehyde increased and reached 2.41 ± 0.05 and 2.55 ± 0.05 U/g after 120 min of treatment, respectively, which was significantly higher than the control (1.90 ± 0.04 U/g).

## Discussion

Essential oils as a replacement for synthetic fungicides are widely used for controlling postharvest diseases [9,20-22]. *p*-Anisaldehyde, primarily isolated from anise, also exhibits strong antifungal activity against a number of yeast and mold strains [14, 23, 24]. Shreaz *et al*. indicated the MIC values of *p*-anisaldehyde for *Candida* (fluconazole-resistant strains and fluconazole-sensitive laboratory strains) ranged from 250 to 600 μg/ml [12]. The MICs of *p*-anisaldehyde for *S. cerevisiae* and *S. aureus* were found to be 256 μg/ml [16] and 2~4 mg/ml [15] respectively. In this research, *p*-anisaldehyde was observed to elicit strong antifungal activity against *P. digitatum* and *P. italicum*, with the MIC and MFC values of both being 2.00 μl/ml, which were much similar with those of the microorganism mentioned above. Furthermore, in vivo assays demonstrated that *p*-anisaldehyde visibly inhibited both the green mold and blue mold development of citrus fruits inoculated with *P. digitatum* and *P. italicum*.

Cell membranes play the vital role of protecting the intracellular substances and maintaining cell viability, and lack of membrane integrity can result in cell death [25, 26]. Many studies have indicated that essential oils such as those extracted from palmarosa, tea tree, thyme, and star anise could inhibit the growth of microorganisms by breaking the membrane integrity [11, 27, 28]. After *p*-anisaldehyde-treatment for 120 min, the mycelia morphology of *P. digitatum* and *P. italicum* was greatly altered. And the SEM results (Fig. 1) showed that the *p*-anisaldehyde-treated mycelia were collapsed and even fractured. The PI staining showed the mycelia of *P. digitatum* and *P. italicum* were stained with PI after *p*-anisaldehyde treatment, suggesting that the membrane integrity of *P. digitatum* and *P. italicum* mycelia was disrupted (Fig. 3).

AKP activity is generally used as the parameter indicating the cell wall integrity [29, 30]. In previous studies, essential oils from tea tree, *Marisela minuta*, and wild blueberry were reported to induce the increase in the AKP activity and thus damaged the cell wall integrity of *Botrytis cinerea*, *Pseudomonas aeruginosa* and various foodborne pathogens [21, 31, 32]. In our previous research, calcofluor white staining and AKP activity were used for the cell wall integrity together. And the experimental results show that these two methods are in agreement with each other [33]. So, AKP activity was used to indicate the cell wall integrity in this research. The results showed that the extracellular AKP activity in 1/2 MIC and MIC *p*-anisaldehyde-treated *P. digitatum* and *P. italicum* mycelia were all significantly (*p* < 0.05) higher than that of control (Fig. 4). Those results suggested that the impairment in the cell wall integrity might be highly involved in the inhibition of *p*-anisaldehyde on *P. digitatum* and *P. italicum*.

In conclusion, our present study indicated that *p*-anisaldehyde exhibited strong antifungal activity against mycelia growths of *P. digitatum* and *P. italicum*. The inhibitory mechanism involved might have to do with the destruction of the cell wall integrity and membrane permeability.

## Figures and Tables

**Table 1:** Effects of *p*-anisaldehyde on mycelial growth of *P. digitatum*.

Concentration (μl/ml)	Inhibitory rate (%)				

	1 d	2 d	3 d	4 d	5 d
0.12	14.3 ± 0.0d	13.2 ± 2.5e	10.5 ± 2.6d	7.3 ± 2.1e	11.5 ± 2.8e
0.25	45.7 ± 4.9c	22.2 ± 2.5d	18.4 ± 2.6d	26.2 ± 2.8d	24.4 ± 2.0d
0.50	62.9 ± 4.9b	64.7 ± 4.4c	46.5 ± 1.5c	34.1 ± 3.2c	31.1 ± 4.3c
1.00	100.0 ± 0.0a	75.7 ± 3.1b	57.9 ± 3.7b	47.9 ± 1.3b	40.7 ± 4.7b
2.00	100.0 ± 0.0a	100.0 ± 0.0a	100.0 ± 0.0a	100.0 ± 0.0a	100.0 ± 0.0a
4.00	100.0 ± 0.0a	100.0 ± 0.0a	100.0 ± 0.0a	100.0 ± 0.0a	100.0 ± 0.0a

^*^The presented data are the means ± standard error of pooled data (*n* = 3). Columns with different letters at each time point indicate significant differences according to LSD test at *p* < 0.05, the same as below.

**Table 2:** Effects of *p*-anisaldehyde on mycelial growth of *P. italicum*.

Concentration (μl/ml)	Inhibitory rate (%)				

	1 d	2 d	3 d	4 d	5 d
0.12	0.0 ± 0.7c	24.1 ± 6.4c	29.8 ± 6.4d	42.4 ± 6.7c	34.5 ± 10.0c
0.25	6.3 ± 1.8c	33.3 ± 11.1c	36.2 ± 8.4cd	36.5 ± 13.3c	28.2 ± 11.6c
0.50	62.5 ± 0.0b	35.2 ± 16.0c	44.7 ± 13.3c	45.8 ± 11.0c	34.0 ± 11.0c
1.00	100.0 ± 0.0a	63.0 ± 3.2b	70.2 ± 3.7b	73.2 ± 3.2b	69.1 ± 1.8b
2.00	100.0 ± 0.0a	100.0 ± 0.0a	100.0 ± 0.0a	100.0 ± 0.0a	100.0 ± 0.0a
4.00	100.0 ± 0.0a	100.0 ± 0.0a	100.0 ± 0.0a	100.0 ± 0.0a	100.0 ± 0.0a

**Table 3:** Green mold incidence in inoculated fruit treated with *p*-anisaldehyde during storage.

Day	Disease incidence (%)		

	CK	MFC	10×MFC
1	0 ± 0	0 ± 0	0 ± 0
2	0 ± 0	0 ± 0	0 ± 0
3	5 ± 5	0 ± 0	0 ± 0
4	30 ± 10	10 ± 10	0 ± 0
5	60 ± 10	60 ± 5	20 ± 5
6	90 ± 5	85 ± 8.6	35 ± 8.6
7	100 ± 0	90 ± 8.6	45 ± 5

^*^The inoculated fruits of CK were sprayed with wax only, and inoculated fruits of MFC or 10×MFC were sprayed with *p*-anisaldehyde wax at 2.0 or 20.0 μl/ml, the same as below.

**Table 4:** Blue mold incidence in inoculated fruit treated with *p*-anisaldehyde during storage.

Day	Disease incidence (%)		

	CK	MFC	10×MFC
1	5 ± 0	0 ± 0	0 ± 0
2	10 ± 5	0 ± 0	0 ± 0
3	10 ± 5	0 ± 0	0 ± 0
4	10 ± 5	0 ± 0	0 ± 0
5	10 ± 5	0 ± 0	0 ± 0
6	10 ± 5	0 ± 0	0 ± 0
7	20 ± 5	0 ± 0	0 ± 0
8	35 ± 0	0 ± 0	0 ± 0
9	45 ± 5	5 ± 5	5 ± 0
10	60 ± 0	10 ± 0	5 ± 0
11	85 ± 5	10 ± 0	5 ± 0
